# The association between oxidative balance score and gallstones in adults: a population-based study

**DOI:** 10.3389/fnut.2025.1534336

**Published:** 2025-03-10

**Authors:** Yuxiao Yang, Jia Wang, Yuan Liu, Jiali Yu, Guanyu Chen, Shiyu Du

**Affiliations:** ^1^Department of Gastroenterology, Peking University China-Japan Friendship School of Clinical Medicine, Beijing, China; ^2^Department of Gastroenterology, China-Japan Friendship Hospital, Beijing, China; ^3^Department of Gastroenterology, The Affiliated Hospital of Qingdao University, Qingdao, China; ^4^Department of Gastroenterology, Chinese Academy of Medical Sciences & Peking Union Medical College, China-Japan Friendship Hospital (Institute of Clinical Medical Sciences), Beijing, China; ^5^Graduate School of Beijing University of Chinese Medicine, Beijing, China

**Keywords:** gallstones, oxidative balance score, diabetes mellitus, mediation analyses, cross-sectional study, National Health and Nutrition Examination Survey

## Abstract

**Purpose:**

Oxidative stress is a significant contributor to the progression of gallstones. However, the combined or independent effects of dietary and lifestyle pro-antioxidants and antioxidants on gallstone formation remain unclear. Our study aims to investigate the potential link between the oxidative balance score (OBS) and the occurrence of gallstones.

**Patients and methods:**

This study utilized data from the National Health and Nutrition Examination Survey (NHANES), conducted in the United States between 2017 and March 2020, identifying 750 gallstone cases among the 7,489 participants. Gallstone status was self-reported. The data in this study were analyzed using a range of statistical techniques, such as Multivariable logistic regression, restricted cubic spline curves (RCS), mediation effects analysis, subgroup analyses and sensitivity analysis.

**Results:**

Using fully adjusted multivariable logistic regression analysis, we identified a significant negative correlation between OBS and the occurrence of gallstones, with an odds ratio (OR) of 0.97 and a 95% confidence interval (CI) of 0.96 to 0.99. Furthermore, participants in the highest quartile of OBS exhibited a 41% reduced risk of gallstones compared to those in the lowest quartile, with an OR of 0.59 (95% CI: 0.45, 0.79) relative to the reference population. Additionally, a linear inverse association between OBS and gallstones was observed. Mediation analysis indicated that diabetes and cardiovascular diseases (CVD) mediated 3.5 and 4% of the association between OBS and gallstones, respectively.

**Conclusion:**

This research suggests that lower OBS levels are associated with a higher susceptibility to gallstone formation, potentially offering a new perspective on clinical strategies for the management and prevention of gallstones.

## Graphical abstract

This cross-sectional study represents an exploration into the relationship between the oxidative balance score and gallstones in the adult population in the United States. Our findings indicate a negative correlation between oxidative balance score levels and gallstones. Of particular note, diabetes and cardiovascular diseases mediated 3.5% and 4% of the association between oxidative balance score and gallstones, respectively.

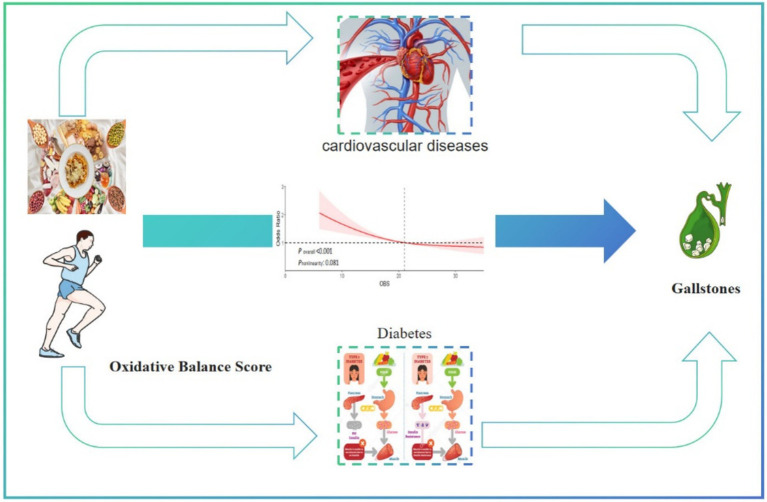

## 1 Introduction

Gallstones are among the most prevalent gastrointestinal disorders worldwide (1). Epidemiological data indicate that cholelithiasis affects between 5 and 25% of adults in Europe and the United States (2). It is estimated that around $62 billion is spent each year in the United States on preventing and treating cholelithiasis (3). Although gallstone disease may not often have evident symptoms, 10% of patients with asymptomatic gallstones will eventually develop symptoms or require treatment within 5 years (4). Gallstones account for 50 to 70% of acute pancreatitis cases, leading to severe abdominal pain and potentially life-threatening infections (5). Moreover, gallstones are a significant risk factor for the development of gallbladder cancer (6). Therefore, it’s critical to identify factors that may be changed or controlled in order to reduce the risk of gallstones.

The oxidative balance score (OBS) integrates dietary and lifestyle factors to assess a person’s oxidative stress level (7). OBS typically suggests a predominance of antioxidants, surpassing pro-oxidants (8). OBS has proven highly useful in epidemiological research, particularly in studies involving chronic diseases, as it measures dietary and lifestyle choices that could negatively impact health (9). Previous research has established a significant connection between OBS and various chronic conditions, such as gastric cancer (10), colorectal cancer (11), type 2 diabetes mellitus (12), cardiovascular disease (CVD) (13). Various foods, nutrients and lifestyle choices are associated with the risk of gallstones, and this association is complex (14). Given these factors, it is plausible to hypothesize that OBS may also influence the risk of developing gallstones. However, no prior investigations have explored the correlations between OBS and gallstones in adults while considering the role of diabetes and CVD.

Hence, this study adopted a cross-sectional study design to explore the associations of OBS and gallstones in US adults who participated in the 2017—March 2020 National Health and Nutrition Examination Survey (NHANES). Further, diabetes and CVD were measured to investigate their intermediary effect and potential mechanisms. Investigating the relationship between OBS and gallstone formation could provide valuable insights into potential preventive strategies, contributing to improved clinical management and reduced incidence of this common gastrointestinal disorder.

## 2 Materials and methods

### 2.1 Study population and design

**Figure 1:**
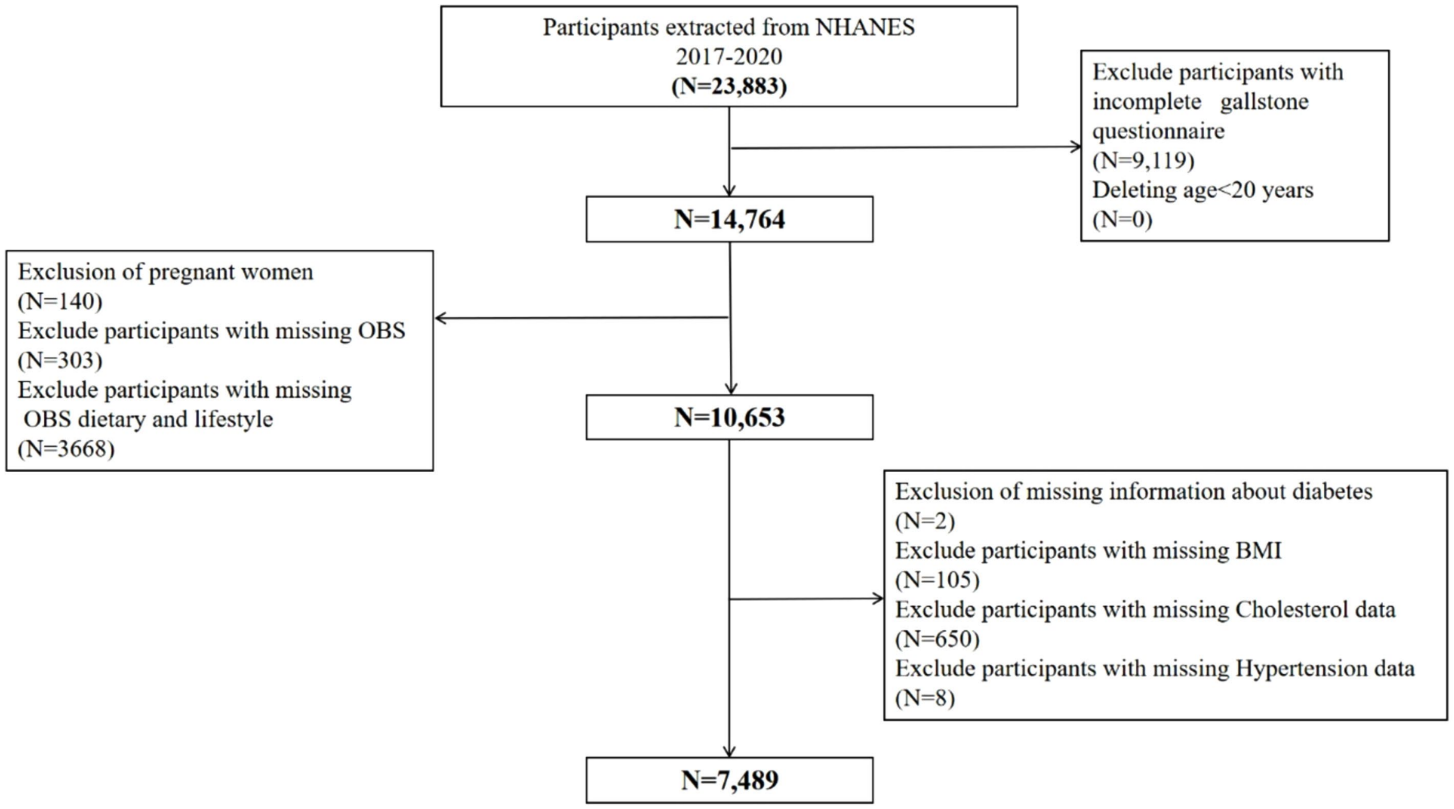
Flowchart of participants selection from NHANES 2017–2020. A total of 23,883 participants recruited between 2017 and 2020 from NHANES were included in the current study, and those younger than age 20 years old (*N* = 0), pregnant (*N* = 140), without data about gallstone questionnaire (*N* = 9,119), OBS (*N* = 303), OBS dietary and lifestyle (*N* = 3,668), diabetes (*N* = 2), BMI (*N* = 105), cholesterol data (*N* = 650), hypertension data (*N* = 8) were excluded. Finally, a total of 7,489 participants were included to evaluate the associations between OBS and gallstones. BMI, body mass index; OBS, oxidative balance score; NHANES, National Health and Nutrition Examination Survey.

The open-source NHANES database, which is formally managed by the Centers for Disease Control and Prevention, provided the data. About 10,000 persons at a time participate in the cross-sectional NHANES survey, which has been updated every 2 years for the past 20 years. Because of the COVID-19 pandemic, only about 60% of the population was polled and finished the survey in 2019–2020. The study data covered the years 2017–2020 since that was when the gallstones questionnaire was only accessible. We eliminated participants under the age of 20 because the questionnaire was only available to people aged 20 and above. We next screened the study population in accordance with the goals of the research, using the comprehensive inclusion and exclusion criteria shown in Figure 1. A total of 7,489 cases were included in this study, of which 750 participants self-reported a history of gallstones.

### 2.2 Ethics approval

All procedures were approved by the National Center for Health Statistics institutional review board and written informed consent was obtained from all participants (available at https://www.cdc.gov/nchs/nhanes/irba98.htm).

### 2.3 Definition of gallstone

Participants were asked whether a doctor had ever diagnosed them with gallstones to determine their presence or absence. A “yes” response indicated that gallstones were present, while a “no” response indicated they were not. This method was used to ensure a clear and straightforward classification of gallstone status among participants.

### 2.4 Definition of OBS

To determine OBS, 16 nutrients and four lifestyle factors were assessed. Based on previous research regarding the relationship between nutrients and oxidative stress, five were identified as pro-oxidants and 15 as antioxidants (15, 16). Quantiles serving as scoring criteria for the 16 nutrients were calculated by averaging nutrient intake from nutrition interviews conducted on the first and second days. The distribution strategy for OBS components is detailed in Table 1. Remaining elements were categorized into three groups based on sex-specific quantiles. Antioxidants were assigned scores from 0 to 2, with higher scores indicating higher antioxidant levels. Conversely, pro-oxidants were scored in reverse, with 0 points assigned to the highest quantile and 2 points to the lowest. Each participant’s total OBS value was then calculated by summing their individual OBS component scores.

**Table 1:** Oxidative balance score allocation scheme.

	Male			Female		
	0	1	2	0	1	2
Dietary OBS components						
Dietary fiber (g/day)	<12.6	<19.7	≥19.7	<10.1	<16.35	≥16.35
Carotene (RE/day)	<99.04	<306.64	≥306.64	<98.25	<383.96	≥383.96
Vitamin B2 (mg/day)	<1.79	<2.69	≥2.69	<1.34	<2.02	≥2.02
Niacin (mg/day)	<20.68	<29.75	≥29.75	<14.528	<21.86	≥21.86
Vitamin B6 (mg/day)	<1.59	<2.40	≥2.40	<1.13	<1.77	≥1.77
Total folate (mcg/day)	<316	<492	≥492	<251	<389	≥389
Vitamin B12 (mcg/day)	<3.36	<6.2	≥19.13	<2.22	<4.22	≥4.22
Vitamin C (mg/day)	<42.5	<113.21	≥113.21	<38.01	<98.5	≥98.5
Vitamin E (ATE) (mg/day)	<5.82	<9.43	≥9.43	<4.54	<7.52	≥7.52
Calcium (mg/day)	<6.46	<1072.5	≥1072.5	<499.5	<849	≥849
Magnesium (mg/day)	<257	<361.28	≥361.28	<187	<283.43	≥283.43
Zinc (mg/day)	<9.75	<15.1	≥15.1	<6.73	<10.73	≥10.73
Copper (mg/day)	<1.12	<1.57	≥1.57	<0.85	<1.28	≥1.28
Selenium (mcg/day)	<94.94	<141.85	≥141.85	<67.83	<99.5	≥99.5
Total fat (g/day)	≥107.42	<107.42	<69.83	≥75.775	<75.775	<50.965
Iron (mg/day)	≥19.165	<19.165	<12.88	≥14.315	<14.315	<9.65
Lifestyle OBS components						
Physical activity (MET-minute/week)	<417.9	<1136.8	≥1136.8	<274	<846	≥846
Alcohol (drinks/day)	≥2 drinks/day	<2 drinks/day	<12 drinks/year	≥1 drinks/day	<1 drinks/day	<12 drinks/year
Body mass index (kg/m^2^)	≥29.16	<29.16	<25.55	≥28.63	<28.63	<23.74
Cotinine (ng/mL)	≥1.08	<1.08	<0.038	≥0.171	<0.171	<0.035

OBS, oxidative balance score.

### 2.5 Ascertainment of covariates

A selection of covariates was made that could potentially act as confounders in the relationship between OBS and gallstones, based on clinical considerations and existing literature. Education levels were categorized into three groups—below high school, high school graduate or GED equivalent, and college or above, as determined by standardized household interviews. Diabetes status was assessed based on one or more of the following criteria: (1) history of insulin use; (2) prior clinical diagnosis of diabetes; (3) use of glucose-lowering medication; or (4) test results indicating a HbA1c level above 6.5% or a fasting blood glucose level exceeding 126 mg/dL (17).

Participants self-reported cardiovascular disease (CVD), which includes conditions such as coronary heart disease, stroke, heart attack, congestive heart failure, and angina. The criteria for including patients with hypertension were: (1) subjects had to provide at least one affirmative response to one of the following questions: “Have you ever been informed more than once that you have hypertension?” or “Have you been diagnosed with hypertension and taken prescribed medication for it?” (2) Subjects were classified as having hypertension if clinical measurements showed an average of three consecutive systolic blood pressure readings of at least 140 mmHg or an average of three consecutive diastolic readings of at least 90 mmHg (18).

### 2.6 Statistical analyses

For this study, we utilized the SURVEY tool to construct weighted populations, as well as samples, strata, and subgroups extracted from the NHANES database. Categorical data were displayed as unweighted counts and weighted proportions, while continuous variables were shown as weighted means and standard errors. To examine the relationship between the OBS and gallstones, we conducted logistic regression analysis using SURVEYLOGISTIC statements. Multivariable regression analyses were performed to adjust for potential confounders identified in single-variable regressions, including age, sex, race, education level, and comorbidities (history of diabetes, hypertension, and CVD). Statistical analyses of categorical variables were conducted using weighted *χ*^2^ tests, while analyses of continuous variables were analyzed with weighted linear regression models to assess differences between groups. Stratified Multivariable regression analysis was employed for subgroup analysis. Additionally, the RCS function was recommended to adjust for continuous exposure, thereby minimizing residual confounding (19). In this study, RCS models were utilized to explore the relationship between the OBS and gallstones both before and after adjustment for confounders. Finally, mediation analyses were performed using the mediation package, and confidence intervals for the mediating effect were assessed using the Bootstrap method to determine the proportion of the mediating effect accounted for by diabetes and CVD. Using these statistical methods, the possible causal relationship between magnesium intake and gallstones can be examined more broadly. Analyses were conducted using the R program and EmpowerStats, considering *p-*values under 0.05 to be statistically significant.

## 3 Results

### 3.1 Baseline characteristics

Table 2 summarizes the baseline characteristics of participants with and without gallstones. Among the 7,489 participants, 750 had gallstones, resulting in a prevalence rate of 10.01%. The average age of participants was 48 years, and the gender distribution was 51% male and 49% female. People with gallstones tended to be older and have lower OBS levels. They also had a significantly higher prevalence of non-Hispanic white ethnicity and were more likely to have diabetes, hypertension, and coronary heart disease (all *p* < 0.05).

**Table 2:** Baseline characteristics of the gallstones group versus the non-gallstones in the NHANES 2017–2020.

Characteristics	Total adults (*N* = 7,489)	No gallstones (*N* = 6,739)	Gallstones (*N* = 750)	*p*-value
Age, years, mean (SE)	47.65 (0.44)	46.66 (0.47)	56.35 (0.76)	**<0.001**
OBS	21.09 (0.22)	21.23 (0.22)	19.90 (0.41)	**<0.001**
OBS dietary	16.45 (0.20)	16.56 (0.20)	15.53 (0.37)	**0.010**
OBS lifestyle	4.64 (0.03)	4.68 (0.03)	4.37 (0.08)	**<0.001**
OBS components				
Fiber intake (g)	16.85 (0.26)	17.05 (0.25)	15.08 (0.49)	**<0.001**
Total fat intake (g)	85.96 (0.66)	87.09 (0.65)	76.08 (1.44)	**<0.001**
Alpha carotene (mcg)	391.16 (16.32)	401.16 (16.44)	304.00 (32.87)	**0.003**
Beta carotene (mcg)	2441.93 (80.33)	2473.60 (87.76)	2165.98 (156.78)	0.100
Riboflavin (mg)	2.13 (0.03)	2.16 (0.03)	1.88 (0.05)	**<0.001**
Niacin (mg)	26.44 (0.32)	27.03 (0.36)	21.31 (0.52)	**<0.001**
Vitamin B6 (mg)	2.19 (0.04)	2.25 (0.04)	1.70 (0.05)	**<0.001**
Total folate (mcg)	380.13 (4.41)	383.90 (4.87)	347.25 (9.11)	**0.001**
Vitamin B12 (mcg)	4.84 (0.09)	4.92 (0.10)	4.08 (0.16)	**<0.001**
Vitamin C (mg)	76.25 (1.13)	77.24 (1.14)	67.61 (3.66)	**0.010**
Vitamin E (ATE) (mg)	9.31 (0.12)	9.47 (0.13)	7.93 (0.23)	**<0.001**
Calcium (mg)	950.90 (10.95)	958.03 (11.30)	888.82 (22.44)	**0.004**
Magnesium (mg)	305.12 (3.51)	309.22 (3.55)	269.36 (6.67)	**<0.001**
Iron (mg)	14.14 (0.14)	14.22 (0.15)	13.42 (0.31)	**0.020**
Zinc (mg)	10.89 (0.11)	11.03 (0.11)	9.66 (0.23)	**<0.001**
Copper (mg)	1.20 (0.01)	1.21 (0.01)	1.09 (0.03)	**<0.001**
Selenium (mcg)	113.28 (0.96)	114.98 (0.99)	98.51 (2.27)	**<0.001**
Carotene (RE)	219.79 (7.23)	222.85 (7.81)	193.16 (13.42)	0.060
Physical activity (MET-minute/week)	5836.03 (193.38)	5921.26 (207.41)	5093.31 (364.78)	**0.050**
Cotinine (ng/mL)	50.94 (3.14)	51.11 (3.45)	49.46 (4.50)	0.770
Gender, *n* (%)				**<0.001**
Female	3,651 (49.07)	3,134 (46.49)	517 (71.62)	
Male	3,828 (50.93)	3,595 (53.51)	233 (28.38)	
Race/ethnicity, *n* (%)				**0.010**
White	2,791 (64.69)	2,449 (63.86)	342 (71.90)	
Black	1,864 (10.81)	1,723 (11.24)	141 (7.02)	
Mexican	892 (8.06)	804 (8.16)	88 (7.18)	
Other	1,932 (16.45)	1,753 (16.74)	179 (13.90)	
Education, *n* (%)				0.110
Grades 0–12	1,106 (8.36)	1,014 (8.56)	92 (6.62)	
High school graduate/GED	1,722 (26.85)	1,530 (26.42)	192 (30.65)	
Some colleges or above	4,651 (64.79)	4,185 (65.02)	466 (62.73)	
Diabetes, (%)				**<0.001**
Yes	1,297 (12.97)	1,086 (11.86)	211 (22.61)	
No	6,182 (87.03)	5,643 (88.14)	539 (77.39)	
Hypertension, (%)				**<0.001**
Yes	2,926 (34.00)	2,515 (32.05)	411 (50.99)	
No	4,553 (66.00)	4,214 (67.95)	339 (49.01)	
Cardiovascular disease				**<0.001**
Yes	777 (8.46)	625 (7.48)	152 (17.02)	
No	6,702 (91.54)	6,104 (92.52)	598 (82.98)	

SE, standard error; OBS, oxidative balance score. All values represented are weighted means ± SE or weighted percentage. *p*-value was calculated by a weighted linear regression model and weighted chi-square test, respectively. *p*-values below a predefined threshold such as 0.05.

### 3.2 Multivariable regression analysis

To analyze the relationship between OBS and gallstones, three models were developed, treating OBS as a continuous and categorical variable (Table 3). In every model, there was a negative association between continuous OBS (encompassing both dietary and lifestyle components) and the risk of gallstones (all *p* < 0.05). After adjusted all covariables, the correlation remained statistically significant (OR = 0.97; 95% CI: 0.95–0.99; *p* < 0.001) even after considering all covariates. When OBS was considered a categorical variable, the risk of gallstones progressively decreased across quartiles in all models (*p* for trend <0.001). In the fully adjusted model, the third quartile (Q3) of OBS showed a 41% lower gallstone risk compared to the first quartile (Q1, reference) [OR (95% CI) = 0.59 (0.45, 0.79), *p* < 0.001]. Similarly, in model 3, both dietary OBS and lifestyle OBS in Q3 compared with Q1 reduced the risk of gallstones by 36 and 49%, respectively (all *p* < 0.01).

**Table 3:** Multivariable regression analysis of oxidative balance score and the risk of gallstone in adult in the NHANES 2017–2020.

	Model 1		Model 2		Model 3	
	OR (95% CI)	*p*	OR (95% CI)	*p*	OR (95% CI)	*p*
OBS	0.97 (0.96, 0.99)	<0.001	0.97 (0.95, 0.98)	<0.001	0.97 (0.95, 0.99)	<0.001
OBS quartile						
Quartile 1	Reference		Reference		Reference	
Quartile 2	0.90 (0.68, 1.19)	0.450	0.78 (0.56, 1.10)	0.150	0.82 (0.59, 1.13)	0.210
Quartile 3	0.62 (0.48, 0.81)	<0.001	0.56 (0.42, 0.75)	<0.001	0.57 (0.44, 0.76)	<0.001
*p* for trend	<0.001		<0.001		<0.001	
OBS dietary	0.98 (0.96, 0.99)	0.010	0.97 (0.95, 0.99)	0.002	0.97 (0.96, 0.99)	0.004
OBS dietary quartile						
Quartile 1	Reference		Reference		Reference	
Quartile 2	0.79 (0.62, 1.00)	0.050	0.69 (0.52, 0.92)	0.010	0.75 (0.59, 0.96)	0.020
Quartile 3	0.67 (0.52, 0.87)	0.003	0.63 (0.47, 0.83)	0.002	0.64 (0.49, 0.84)	0.002
*p* for trend	0.003		0.002		0.002	
OBS lifestyle	0.85 (0.79, 0.92)	<0.001	0.82 (0.74, 0.91)	<0.001	0.87 (0.79, 0.95)	0.003
OBS lifestyle quartile						
Quartile 1	Reference		Reference		Reference	
Quartile 2	0.63 (0.50, 0.80)	<0.001	0.57 (0.43, 0.76)	<0.001	0.56 (0.41, 0.76)	<0.001
Quartile 3	0.51 (0.38, 0.68)	<0.001	0.46 (0.32, 0.65)	<0.001	0.51 (0.36, 0.71)	<0.001
*p* for trend	<0.001		<0.001		<0.001	

OBS, oxidative balance score; OR, odds ratio; 95% CI, 95% confidence interval; CVD, cardiovascular disease. Model 1 was an unadjusted model. Model 2 was adjusted for age, sex, race, and education level. Model 3 was adjusted for age, sex, race, education level, history of diabetes, hypertension, and CVD.

### 3.3 Non-linear relationship exploration

**Figure 2:**
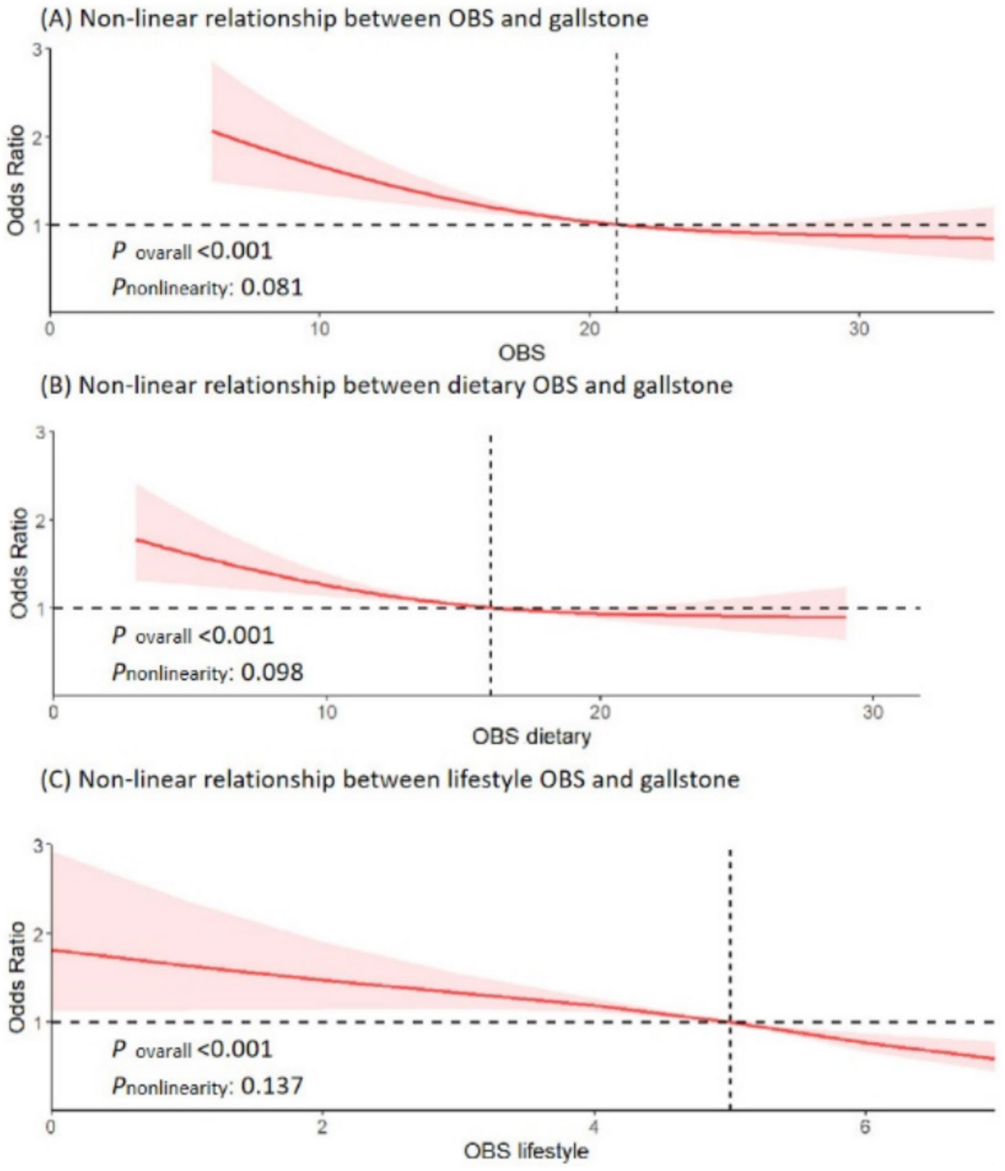
Non-linear relationship of all oxidative balance score with gallstone. **(A)** Non-linear relationship between OBS and gallstone. **(B)** Non-linear relationship between dietary OBS and gallstone. **(C)** Non-linear relationship between lifestyle OBS and gallstone. OBS, oxidative balance score; OR, odds ratio; 95% CI, 95% confidence interval.

We explored the non-linear relationship between OBS and risk of gallstones using RCS models. OBS, along with dietary OBS, and lifestyle OBS all showed a linear association with risk of gallstones (all *p* for overall <0.001, *p* non-linearity >0.05) (Figure 2). Interestingly, there was a significant negative association between OBS (both dietary and lifestyle OBS) and gallstones risk.

### 3.4 Mediation analysis

**Figure 3:**
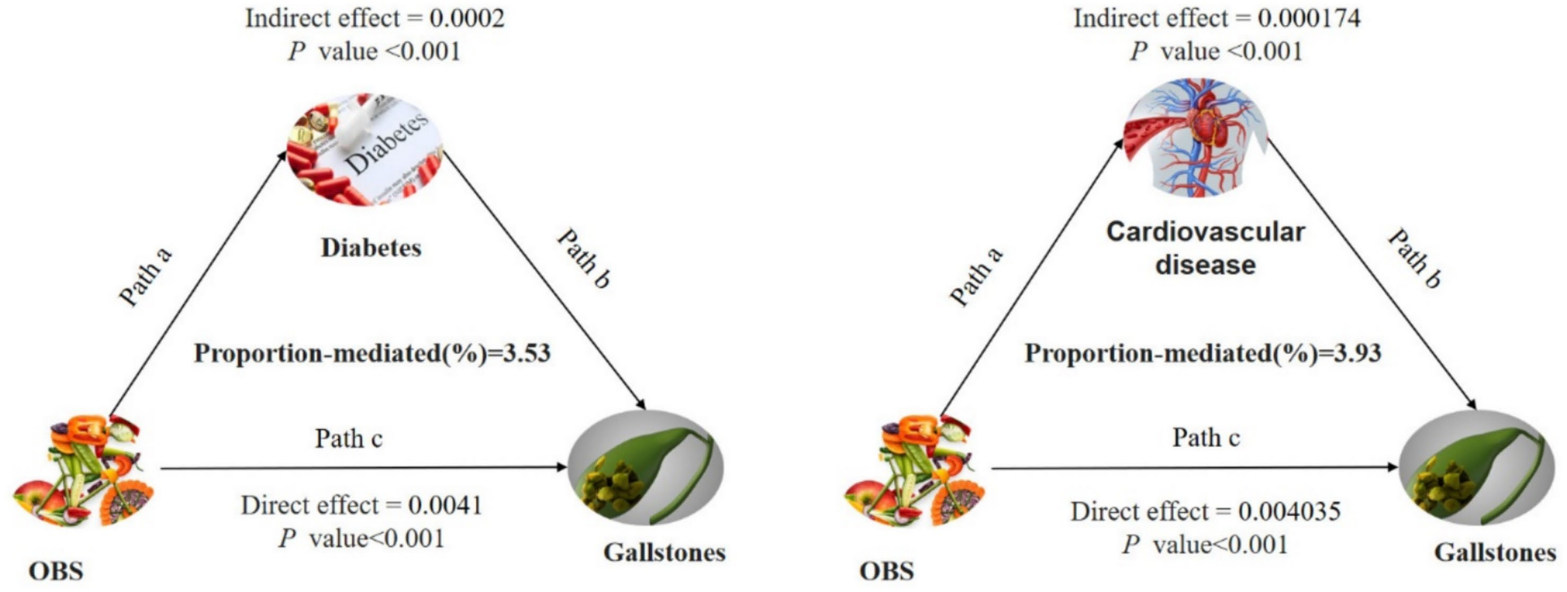
Path diagram of the mediation analysis model for the 7,489 adult participants in the 2017–2020 National Health and Nutrition Survey. In the mediation analysis, OBS is defined as the exposure factor; gallstones are defined as the outcome; and diabetes and cardiovascular diseases is defined as the mediator. Path a represents the regression coefficient for the association of OBS with diabetes or cardiovascular diseases. Path b represents the regression coefficient for the association of diabetes or cardiovascular diseases with gallstones. Path c denotes the simple total effect of OBS versus gallstones, unadjusted for diabetes or cardiovascular diseases. OBS, oxidative balance score.

Conduct mediation analyses to evaluate whether diabetes and CVD mediate the relationship between OBS and gallstone occurrence. Figure 3 illustrates the model and pathway for the mediation analysis. OBS had a significant direct effect on gallstone occurrence, even in the presence of each mediating variable (all *p* < 0.001). After accounting for all potential confounders, indirect mediation effects were identified for diabetes and CVD. The average mediated proportions of diabetes and CVD were 3.5 and 4%, respectively (all *p* < 0.001) (Table 4).

**Table 4:** Mediation analysis of the relationship between OBS and gallstones.

Independent variable	Mediator	Direct effect (average)	*p*	Mediation effect (average)	*p*	Proportion-mediated (average)	*p*
OBS	Diabetes	−0.0041 (−0.0063, −0.0023)	<0.001	−0.0002 (−0.0003, −0.0063)	<0.001	0.0353 (0.0159, 0.0725)	<0.001
OBS	Cardiovascular diseases	−0.004035 (−0.006282, −0.00229)	<0.001	−0.000174 (−0.0003, −0.0021)	<0.001	0.0393 (0.0187, 0.0778)	<0.001

OBS, oxidative balance score.

### 3.5 Subgroup analysis

**Figure 4:**
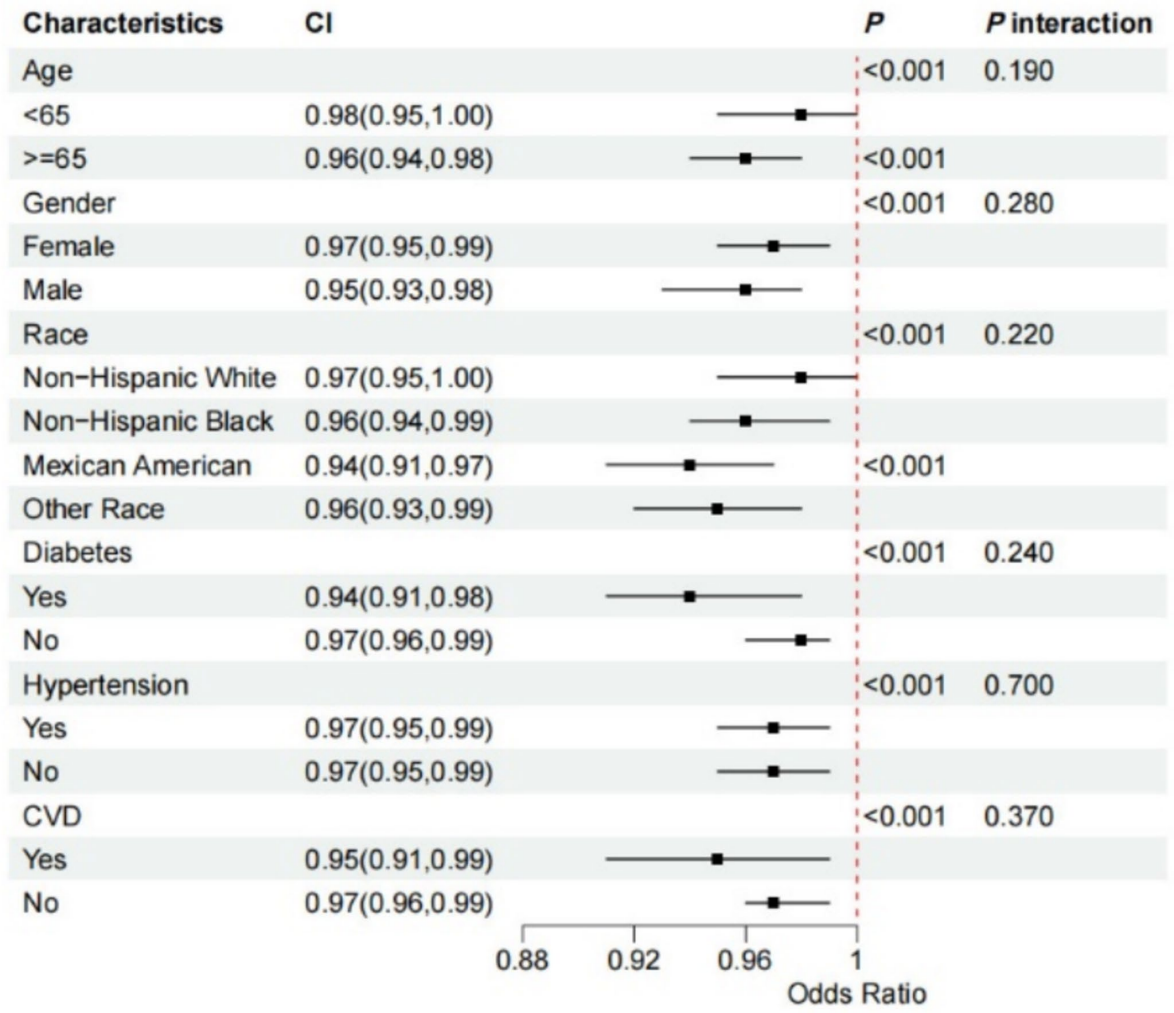
Forest plot for stratified analysis. The participants were classified into quintiles and we used the stratified multivariable regression analysis. The solid dot and the line represent estimates of hazard ratios and the 95% confidence intervals, respectively. Covariates included age, sex (male or female), race (non-Hispanic White, non-Hispanic Black, Mexican American, or other), diabetes, hypertension, and CVD. 95% CI, 95% confidence interval; CVD, cardiovascular diseases.

We conducted a subgroup analysis to determine if the association between OBS and gallstone risk was consistent across different subgroups. Notably, the relationship between OBS and increased gallstone risk was particularly strong among participants who were over 60 years old, male, Mexican, diabetes and CVD (all *p*-values <0.05). The interaction test revealed that the *p*-value for interaction was >0.05 across subgroups, including age, sex, race, diabetes, hypertension, and CVD, indicating consistency in our findings across all subgroups (Figure 4).

### 3.6 Sensitivity analysis

To confirm the robustness of our results, we conducted a sensitivity analysis. Given that OBS comprises multiple components, we examined the relationship between each component and gallstone incidence (Supplementary Table S1). Our analysis revealed that, among the 20 components, only BMI was positively correlated with gallstone occurrence. Specifically, individuals with higher BMI were more likely to develop gallstones. Furthermore, similar results were obtained after adjusting for coffee intake and PIR, as shown in Supplementary Tables S2, S3. These findings underscore the robustness and stability of our results.

## 4 Discussion

Analyzing data from a nationally representative US cross-sectional study spanning 20 years, our findings support the hypothesis that higher OBS scores are linearly associated with a reduced risk of gallstones, with similar effects observed for both dietary and lifestyle OBS independently. At the same time, diabetes and CVD partially mediated the relationship between OBS and gallstones. This finding underscores the importance of focusing on blood glucose management and enhancing care for diabetic and cardiovascular patients while also regulating diet and lifestyle to prevent gallstones.

Our results on gallstones and OBS were consistent with the majority of existing research, demonstrating dependability. Numerous substances included in the OBS diet have previously been shown to possess antioxidant properties. Research findings indicate that a higher intake of dietary fiber may help prevent gallstones by reducing intestinal transit time (20). Furthermore, dietary fibers may reduce the persistence of colonic bacteria, leading to a decrease in the production of secondary bile acids, such as deoxycholate. Consequently, intake of dietary fiber can lower the formation of gallstones (21, 22). Research indicates that a diet rich in meat, animal fats, and fried foods elevates the risk of gallstones. Conversely, consuming fruits, vegetables, nuts, fish, monounsaturated fatty acids (MUFA), and n-3 fatty acids may offer protective benefits against gallstones (23). In addition, energy intake, along with macronutrients and micronutrients including niacin, vitamin E, calcium and magnesium may reduce oxidative stress in gallstone patients (24–26).

Many studies have described the impact of lifestyle on gallstone formation, and these lifestyle factors were included in our OBS components. Prior studies found that individuals with a higher physical activity index were at a reduced risk for gallbladder disease, and participating in vigorous exercise more than twice a week was linked to a decreased likelihood of developing gallstones (27, 28). The protective effect of exercise is thought to stem from its ability to guard against central and general obesity (29, 30), as well as its role in increasing plasma cholecystokinin (31) and vagal tone (32), which together stimulate gallbladder contraction and emptying. Oxidative stress is independently linked to obesity, with oxidative stress levels increasing alongside BMI and age, leading to progressively impaired antioxidant status (33). As BMI increases, the risk of symptomatic cholelithiasis also rises, driven by a combination of metabolic factors, dyslipidemia, gallbladder stasis, changes in bile composition, and cholesterol crystallization, ultimately leading to cholesterol-laden gallstones (34). In summary, previous studies predominantly focused on the influence of single dietary components or isolated lifestyle factors on gallstone formation. However, examining individual components or factors might not sufficiently explain their effects of antioxidants on the body. In contrast, our study used OBS as a measure of the overall balance between oxidation and antioxidation, providing a more comprehensive reflection of oxidative stress levels in body. Future larger prospective researches are required to validate our findings further.

Our mediation analysis indicates that CVD and diabetes influence the connection between OBS and gallstones. Previous research has shown a correlation between diabetes mellitus, CVD, and gallstones, suggesting that individuals with these conditions have a higher likelihood of developing gallstones compared to the general population. This risk is especially pronounced in those with poorly controlled blood glucose levels and cardiovascular-related risk factors (35–37). While the exact mechanism by which OBS affects susceptibility to gallstones remains uncertain, oxidative stress could play a significant role. First, buildup of reactive oxygen species (ROS) disrupts the intracellular balance between antioxidants and pro-oxidants, thereby driving resistance to insulin and the peroxidation of lipids via various mechanisms. This disruption adversely affects glucose and lipid metabolism, thus elevating the likelihood of cardiovascular diseases (38, 39). Secondly, ROS contributes to decreased blood flow to the gallbladder and decreased peristaltic capacity through damage to the endothelium, dysfunction and remodeling of blood vessels, and stimulation of the sympathetic nervous system. Consequently, prolonged stasis of bile in the gallbladder raises bile saturation, which can lead to cholecystitis and gallstone formation (40–42). Moreover, decreased responsiveness of gallbladder smooth muscle cells to cholecystokinin, or a reduction in the quantity of cholecystokinin receptors within the gallbladder wall, may also play a role. In diabetic patients, insulin-related signaling pathways become aberrant primarily due to reduced punctate signaling of the insulin receptor in human hepatocytes and elevated levels of ROS, which decrease the functional activity of insulin receptor clusters in cells that are otherwise insulin-sensitive (43–45). Further studies are needed to explore the specific mechanisms involved. While type 2 diabetes and CVD were chosen due to their well-established links to both oxidative stress and gallstone disease, other chronic conditions may indeed play a role. We acknowledge the potential mediating effects of additional conditions, such as metabolic syndrome and gastrointestinal cancers, and suggested their inclusion in future research.

The study has several strengths and weaknesses. Notably, this is the initial study to evaluate the relationship between OBS and gallstones in US adults. Furthermore, due to the stratified and multistage design of the NHANES data, our findings possess a high degree of generalizability within the mobile population. Additionally, we attempted to control for a variety of confounders by utilizing a comprehensive questionnaire and extensive adjustments for variables. Despite its strengths, this study has several limitations. First, the cross-sectional nature of the present study precludes effective assessment of causality. While we accounted for potential confounders, the influence of unknown factors cannot be entirely dismissed. Second, the diagnosis of gallstones was based only on responses to a medical health questionnaire and lacked a more precise imaging diagnosis. Third, mediation analysis is based on causal hypotheses, where we generally assume that the mediator occurs after the exposure. However, in a cross-sectional study, the temporal relationship cannot be verified. Third, survey data from NHANES were based on questionnaires, which means that recall bias may exist. Despite these limitations, this paper still reveals for the first time the relationship between OBS and gallstone prevalence and provides strong support for OBS as a predictor of gallstone development. As a next step, we will conduct a multicentre prospective cohort study and construct relevant clinical prediction models to further explore the impact of OBS on the prevalence of gallstones in the real world.

## 5 Conclusion

Elevated OBS levels are linked to a decreased risk of gallstone development, with diabetes and cardiovascular diseases acting as mediating factors. The potential role of oxidative stress in gallstone prevention underscores its significance in clinical and public health practices.

## Data Availability

The original contributions presented in the study are included in the article/Supplementary material, further inquiries can be directed to the corresponding author.
