# Supplementary material for: The association between oxidative balance score and gallstones in adults: a population-based study
Source: Front Nutr. 2025 Mar 10;12:1534336. doi: 10.3389/fnut.2025.1534336 (PMC11932657; doi:10.3389/fnut.2025.1534336)
Supplement: Supplementary file 1 [file Table_1.pdf]

TITLE PAGE：

Type of the Study: Original Research Article

Lower oxidative balance score levels were associated with a higher risk of gallstones in US Adults

Author information:

Yuxiao Yang[1,5#], Jia Wang [2#], Yuan Liu [3,5], Jiali Yu [3,5], Guanyu Chen [3,5], Shiyu Du[5*]

1 Department of Gastroenterology, Peking University China-Japan Friendship School of Clinical Medicine, Beijing, China.

2 Department of Gastroenterology, The Affiliated Hospital of Qingdao University, Qingdao, Shandong, China.

3 Graduate School of Beijing University of Chinese Medicine, Beijing, China.

4 Department of Gastroenterology, Chinese Academy of Medical Sciences & Peking Union Medical College, China-Japan Friendship Hospital (Institute of Clinical Medical Sciences), Beijing, China.

5 Department of Gastroenterology, China-Japan Friendship Hospital, Beijing, China.

[#] Yuxiao Yang and Jia Wang contributed equally to this work as co-first authors.

[*]**Corresponding author:**

Shiyu Du, MD

Department of Gastroenterology, China-Japan Friendship Hospital, Beijing, China.

Tel: 86-17852720179, Email: dushiyu1975@126.com

Supplementary Table S1 Each oxidative balance score component and gallstone incidence

	Crude Model		Model 1		Model 2	
	OR (95% CI)	*P*	OR (95% CI)	*P*	OR (95% CI)	*P*
Dietary fiber (g/d)	0.97(0.96,0.99)	<0.001	0.98(0.96,0.99)	0.010	0.98(0.96,0.99)	0.010
Total fat (g/d)	0.99(0.99,0.99)	<0.001	1.00(0.99,1.00)	0.010	1.00(0.99,1.00)	0.010
Riboflavin (mg/d)	0.79(0.70,0.90)	<0.001	0.89(0.78,1.02)	0.090	0.89(0.78,1.02)	0.080
Niacin (mg/d)	0.96(0.95,0.97)	<0.001	0.98(0.97,0.99)	0.001	0.98(0.97,0.99)	0.001
Vitamin B6 (mg/d)	0.68(0.60,0.78)	<0.001	0.79(0.69,0.91)	0.002	0.79(0.69,0.91)	0.002
Total folate (mcg/d)	1.00(1.00,1.00)	0.004	1.00(1.00,1.00)	0.710	1.00(1.00,1.00)	0.780
Vitamin B12 (mcg/d)	0.94(0.90,0.97)	<0.001	0.97(0.94,1.00)	0.080	0.97(0.94,1.00)	0.060
Vitamin C (mg/d)	1.00(1.00,1.00)	0.030	1.00(1.00,1.00)	0.050	1.00(1.00,1.00)	0.060
Vitamin E (ATE) (mg/d)	0.94(0.92,0.97)	<0.001	0.95(0.93,0.98)	0.001	0.95(0.93,0.98)	0.001
Calcium (mg/d)	1.00(1.00,1.00)	0.010	1.00(1.00,1.00)	0.810	1.00(1.00,1.00)	0.910
Magnesium (mg/d)	1.00(1.00,1.00)	<0.001	1.00(1.00,1.00)	0.004	1.00(1.00,1.00)	0.010
Iron (mg/d)	0.98(0.97,1.00)	0.030	1.01(0.99,1.02)	0.510	1.01(0.99,1.02)	0.490
Zinc (mg/d)	0.95(0.93,0.97)	<0.001	0.98(0.96,1.00)	0.080	0.98(0.96,1.00)	0.070
Copper (mg/d)	0.64(0.48,0.86)	0.004	0.74(0.53,1.03)	0.070	0.75(0.53,1.06)	0.100
Selenium (mcg/d)	0.99(0.99,1.00)	<0.001	1.00(0.99,1.00)	0.040	1.00(0.99,1.00)	0.040
Alcohol (drinks/d)	0.99(0.98,0.99)	<0.001	0.99(0.99,1.00)	0.020	0.99(0.99,1.00)	0.040
Body mass index (kg/m^2)	1.07(1.06,1.08)	<0.001	1.08(1.06,1.09)	<0.001	1.07(1.06,1.09)	<0.001
Physical activity (MET minute/week)	1.00(1.00,1.00)	0.070	1.00(1.00,1.00)	0.120	1.00(1.00,1.00)	0.060
Cotinine (ng/mL)	1.00(1.00,1.00)	0.770	1.00(1.00,1.00)	0.020	1.00(1.00,1.00)	0.020

Carotene (RE/d)	1.00(1.00,1.00)	0.100	1.00(1.00,1.00)	0.010	1.00(1.00,1.00)	0.010

Abbreviations: OBS, oxidative balance score; OR, odds ratio; 95% CI, 95% confidence interval;

Supplementary table S2 Multivariate regression analysis of oxidative balance score and risk of gallstones with adjustment for coffee intake

	Model 1		Model 2		Model 3	
	OR (95% CI)	*P*	OR (95% CI)	*P*	OR (95% CI)	*P*
OBS	0.97(0.96,0.99)	<0.001	0.97(0.95,0.98)	<0.001	0.97(0.95,0.98)	<0.001
OBS Quartile						
Quartile 1	reference		reference		reference	
Quartile 2	0.90(0.68,1.19)	0.460	0.79(0.56,1.10)	0.150	0.82(0.59,1.14)	0.220
Quartile 3	0.62(0.48,0.81)	<0.001	0.56(0.42,0.74)	<0.001	0.58(0.44,0.76)	<0.001
P for trend	<0.001		<0.001		<0.001	
OBS Dietary	0.98(0.96,0.99)	0.010	0.97(0.95,0.99)	0.002	0.97(0.96,0.99)	0.003
OBS Dietary Quartile						
Quartile 1	reference		reference		reference	
Quartile 2	0.79(0.62,1.01)	0.060	0.70(0.52,0.93)	0.020	0.73(0.55,0.96)	0.030
Quartile 3	0.67(0.52,0.87)	0.004	0.63(0.47,0.83)	0.002	0.64(0.49,0.84)	0.002
P for trend	0.004		0.002		0.002	
OBS Lifestyle	0.85(0.78,0.91)	<0.001	0.81(0.73,0.90)	<0.001	0.83(0.74,0.92)	0.001
OBS Lifestyle Quartile						
Quartile 1	reference		reference		reference	
Quartile 2	0.63(0.50,0.80)	<0.001	0.56(0.41,0.75)	<0.001	0.55(0.40,0.76)	<0.001
Quartile 3	0.51(0.38,0.68)	<0.001	0.45(0.32,0.64)	<0.001	0.50(0.36,0.70)	<0.001
P for trend	<0.001		<0.001		<0.001	

Model 1 was an unadjusted model.

Model 2 was adjusted for age, sex, race, and education level.

Model 3 was adjusted for age, sex, race, education level, diabetes, hypertension, and CVD.

Abbreviations: OBS, oxidative balance score; OR, odds ratio; 95% CI, 95% confidence interval; CVD, Cardiovascular disease.

Supplementary table S3 Multivariate regression analysis of oxidative balance score and risk of gallstones with adjustment for Poverty-to-income ratio.

	Model 1		Model 2		Model 3	
	OR (95% CI)	*P*	OR (95% CI)	*P*	OR (95% CI)	*P*
OBS	0.97(0.95,0.99)	0.002	0.97(0.95,0.98)	<0.001	0.97(0.95,0.98)	<0.001
OBS Quartile						
Quartile 1	reference		reference		reference	
Quartile 2	0.93(0.70,1.22)	0.580	0.81(0.58,1.14)	0.220	0.84(0.61,1.16)	0.280
Quartile 3	0.61(0.46,0.82)	0.001	0.57(0.42,0.78)	<0.001	0.58(0.43,0.79)	0.001
P for trend	<0.001		<0.001		<0.001	
OBS Dietary	0.98(0.96,0.99)	0.010	0.97(0.95,0.99)	0.010	0.97(0.96,0.99)	0.010
OBS Dietary Quartile						
Quartile 1	reference		reference		reference	
Quartile 2	0.84(0.65,1.09)	0.180	0.74(0.54,1.01)	0.050	0.77(0.57,1.03)	0.080
Quartile 3	0.67(0.52,0.87)	0.004	0.65(0.48,0.87)	0.010	0.66(0.49,0.88)	0.010
P for trend	0.002		0.002		0.002	
OBS Lifestyle	0.83(0.76,0.90)	<0.001	0.80(0.72,0.89)	<0.001	0.82(0.74,0.90)	<0.001
OBS Lifestyle Quartile						
Quartile 1	reference		reference		reference	
Quartile 2	0.61(0.48,0.78)	<0.001	0.54(0.40,0.73)	<0.001	0.54(0.39,0.74)	<0.001
Quartile 3	0.47(0.35,0.64)	<0.001	0.43(0.31,0.60)	<0.001	0.47(0.35,0.64)	<0.001
P for trend	<0.001		<0.001		<0.001	

Model 1 was an unadjusted model.

Model 2 was adjusted for age, sex, race, and education level.

Model 3 was adjusted for age, sex, race, education level, diabetes, hypertension, and CVD.

Abbreviations: OBS, oxidative balance score; OR, odds ratio; 95% CI, 95% confidence interval; CVD, Cardiovascular disease; PIR, Poverty-to-income ratio.
